# Retraction Note: Proteomic characterization identifies clinically relevant subgroups of soft tissue sarcoma

**DOI:** 10.1038/s41467-026-76422-6

**Published:** 2026-08-06

**Authors:** Shaoshuai Tang, Yunzhi Wang, Rongkui Luo, Rundong Fang, Yufeng Liu, Hang Xiang, Peng Ran, Yexin Tong, Mingjun Sun, Subei Tan, Wen Huang, Jie Huang, Jiacheng Lv, Ning Xu, Zhenmei Yao, Qiao Zhang, Ziyan Xu, Xuetong Yue, Zixiang Yu, Sujie Akesu, Yuqin Ding, Chen Xu, Weiqi Lu, Yuhong Zhou, Yingyong Hou, Chen Ding

**Affiliations:** 1 State Key Laboratory of Genetic Engineering and Collaborative Innovation Center for Genetics and Development, School of Life Sciences, Institutes of Biomedical Sciences, Human Phenome Institute, Department of General Surgery, Zhongshan Hospital, Fudan University, Shanghai, 200433 China; 2 Department of Pathology, Zhongshan Hospital, Fudan University, Shanghai, China; 3 Shanghai Institute of Medical Imaging, Shanghai, China; 4 Department of General Surgery, Zhongshan Hospital, Fudan University, Shanghai, China; 5 Department of Medical Oncology, Zhongshan Hospital, Fudan University, Shanghai, China

Retraction Note to: *Nature Communications* 10.1038/s41467-024-45306-y, published online 15 February 2024

The authors have retracted this article. After publication, concerns were raised regarding potential similarities between Figs. 2F-1 and 2J-4; Figs. 2I-4 and 2J-1; Figs. 2I-1 and 3L-1; Figs. 2I-3 and 2J-2; Fig. 2I-4 and Supplementary Fig. 10B-4; Figs. 2J-1 and 3L-1; Figs. 2J-3 and 3L-2; Supplementary Figs. 7C-25 and 7C-5; Supplementary Figs. 10B-1 and 10B-3; Supplementary Figs. 14D-7 and 14D-8; Supplementary Figs. 14D-11 and 14D-12; and Fig. 3H-1 in this article and Fig. 5G-3 of^1^. The Editors therefore no longer have confidence in the reliability of the data reported in the article.

Yunzhi Wang has stated on behalf of all authors that they agree with this retraction.
